# Supplementary material for: Interpretable machine learning for high-dimensional trajectories of aging health
Source: PLoS Comput Biol. 2022 Jan 10;18(1):e1009746. doi: 10.1371/journal.pcbi.1009746 (PMC8782527; doi:10.1371/journal.pcbi.1009746)
Supplement: S1 Text — Supplemental derivations, alternate models, and tables. Table A. Variables used from the ELSA dataset. Background variables are only used at the first time-step, as ut0. Longitudinal variables are predicted in yt. All variables are z-scored; additional transformations before z-scoring are indicated. Table B. Activities of daily living (ADL) and Instrumental activities of daily living (IADL) from the ELSA dataset, for a total of 10 ADL and 13 IADL. Table C. Neural network architectures used in the DJIN model, as described in Fig 1 and “Network architecture and Hyperparameters” of the methods. The health variables yt0 are size N = 29, the health variable observed mask ot0 is of size N = 29, and the background health variables ut0 with appended missing mask are of size B + 17 = 36. (PDF) [file pcbi.1009746.s001.pdf]

Supplemental Information for: Interpretable machine learning for high-dimensional trajectories of aging health

Spencer Farrell, Arnold Mitnitski, Kenneth Rockwood, and Andrew D. Rutenberg

I. DERIVING THE VARIATIONAL LOSS

We denote health variables observed at age t_k by $\mathbf{y}_{t_k}$, the background information at baseline by $\mathbf{u}_{t_0}$, the model health variable predictions by $\mathbf{x}(t_k)$, the latent variables for imputation/generation by $\mathbf{z}$, the age of death or last censoring age by a, the censoring indicator by c, parameters by $\boldsymbol{\theta}$, and variational parameters by $\boldsymbol{\phi}$.

To fit the model, we minimize the KL-divergence between the approximate posterior and the true posterior. This is equivalent to maximizing a lower bound to the model evidence. Starting with the model evidence,

$$
\begin{aligned}
&\log p(\{\mathbf{y}_{t_k}\}_k|\mathbf{u}_{t_0},\{\mathbf{o}_{t_k}\}_k,a,c) \qquad (1)\\
&= \log \int d\boldsymbol{\theta} d\mathbf{z} d\mathbf{x}_0 dt\, p(\boldsymbol{\theta})p(\mathbf{z})p(\mathbf{x}_0|\mathbf{z},\mathbf{u}_{t_0},t_0)p(\mathbf{x}(t)|\mathbf{x}_0,\mathbf{u}_{t_0},t)p(a,c|\mathbf{x}(t),\mathbf{u}_{t_0},t)\prod_{k=0}^{K}p(\mathbf{y}_{t_k}|\mathbf{x}(t_k),\mathbf{o}_{t_k})\\
&= \log \int d\boldsymbol{\theta} d\mathbf{z}\, p(\boldsymbol{\theta})p(\mathbf{z})\int d\mathbf{x}_0\, p(\mathbf{x}_0|\mathbf{z},\mathbf{u}_{t_0},t_0)\int dt\, p(\mathbf{x}(t)|\mathbf{x}_0,\mathbf{u}_{t_0},t)p(a,c|\mathbf{x}(t),\mathbf{u}_{t_0},t)\prod_{k=0}^{K}p(\mathbf{y}_{t_k}|\mathbf{x}(t_k),\mathbf{o}_{t_k})\\
&= \log \int d\boldsymbol{\theta} d\mathbf{z}\, p(\boldsymbol{\theta})p(\mathbf{z})\frac{q(\mathbf{z}|\mathbf{y}_{t_0},\mathbf{u}_{t_0},\mathbf{o}_{t_0},t_0)q(\boldsymbol{\theta})}{q(\mathbf{z}|\mathbf{y}_{t_0},\mathbf{u}_{t_0},\mathbf{o}_{t_0},t_0)q(\boldsymbol{\theta})}\int d\mathbf{x}_0\, p(\mathbf{x}_0|\mathbf{z},\mathbf{u}_{t_0},t_0)\int dt\, p(\mathbf{x}(t)|\mathbf{x}_0,\mathbf{u}_{t_0},t)\frac{q(\mathbf{x}(t)|\mathbf{x}_0,\mathbf{u}_{t_0},t)}{q(\mathbf{x}(t)|\mathbf{x}_0,\mathbf{u}_{t_0},t)}p(a,c|\mathbf{x}(t),\mathbf{u}_{t_0},t)\\
&\times \prod_{k=0}^{K}p(\mathbf{y}_{t_k}|\mathbf{x}(t_k),\mathbf{o}_{t_k})\\
&= \log \mathbb{E}_{\mathbf{z},\boldsymbol{\theta}\sim q}\Big[\frac{p(\mathbf{z})}{q(\mathbf{z}|\mathbf{y}_{t_0},\mathbf{u}_{t_0},\mathbf{o}_{t_0},t_0)}\mathbb{E}_{\mathbf{x}_0|\mathbf{z}\sim p,\mathbf{x}(t)|\mathbf{x}_0\sim q}\Big[\int_{t_0}^{a}\frac{p(\mathbf{x}(t)|\mathbf{x}_0,\mathbf{u}_{t_0},t)}{q(\mathbf{x}(t)|\mathbf{x}_0,\mathbf{u}_{t_0},t)}p(a,c|\mathbf{x}(t),\mathbf{u}_{t_0},t)dt\prod_{k=0}^{K}p(\mathbf{y}_{t_k}|\mathbf{x}(t_k),\mathbf{o}_{t_k})\Big]\Big],
\end{aligned}
$$

where we have introduced the approximate posteriors q. Using Jensen's Inequality we move the logarithm into the expectations, and define this lower bound as the objective function,

$$
\begin{aligned}
&\mathcal{L}(\boldsymbol{\phi}) = \mathbb{E}_{\boldsymbol{\theta},\mathbf{z}\sim q,\ \mathbf{x}_0|\mathbf{z}\sim p,\ \mathbf{x}(t)|\mathbf{x}_0\sim q}\left[\int_{t_0}^{a}\log\frac{p(\boldsymbol{\theta})p(\mathbf{z})p(\mathbf{x}(t)|\mathbf{x}_0,\mathbf{u}_{t_0},t)p(\mathbf{y}_t|\mathbf{x}(t),\mathbf{o}_{t_k})p(a,c|\mathbf{x}(t),\mathbf{u}_{t_0},t_0)}{q(\mathbf{z}|\mathbf{y}_{t_0},\mathbf{u}_{t_0},\mathbf{o}_{t_0},t_0)q(\boldsymbol{\theta})q(\mathbf{x}(t)|\mathbf{x}_0,\mathbf{u}_{t_0},t)}dt\right] \qquad (2)\\
&= \mathbb{E}\left[\sum_k \log p(\mathbf{y}_{t_k}|\mathbf{x}(t_k),\mathbf{o}_{t_k}) + \int_{t_0}^{a}\Big\{\log p(a,c|\mathbf{x}(t),\mathbf{u}_{t_0},t) + \log p(\mathbf{x}(t)|\mathbf{x}_0,\mathbf{u}_{t_0},t) - \log q(\mathbf{x}(t)|\mathbf{x}_0,\mathbf{u}_{t_0},t)\Big\}dt\right]\\
&- KL(q(\boldsymbol{\theta})||p(\boldsymbol{\theta})) - KL(q(\mathbf{z}|\mathbf{y}_{t_0},\mathbf{u}_{t_0},\mathbf{o}_{t_0},t_0)||p(\mathbf{z}))\\
&= \mathbb{E}\Bigg[\sum_k \mathbf{o}_{t_k}\odot\log\mathcal{N}(\mathbf{y}_{t_k}|\mathbf{x}(t_k),\boldsymbol{\sigma}_{\mathbf{y}}) + (1-c)\big[\log\lambda(a|\mathbf{x}(t),\mathbf{u}_{t_0},t_0) + \log S(a|\mathbf{x}(t),\mathbf{u}_{t_0},t_0)\big] + \int_{t_0}^{a} c\log S(t|\mathbf{x}(t),\mathbf{u}_{t_0},t_0)dt\\
&+ \int_{a}^{a_{\max}}(1-c)\log\left(1 - S(t|\mathbf{x}(t),\mathbf{u}_{t_0},t_0)\right)dt - \frac{1}{2}\int_{t_0}^{a}\Big|\Big|\boldsymbol{\sigma}_x^{-1}\odot\big(\mathbf{W}\mathbf{x} + \mathbf{f}(\mathbf{x}(t),\mathbf{u}_{t_0},t) - \mathbf{g}(\mathbf{x}(t),\mathbf{u}_{t_0},t)\big)\Big|\Big|^2 dt\Bigg]\\
&- KL(q(\boldsymbol{\theta})||p(\boldsymbol{\theta})) - KL(q(\mathbf{z}|\mathbf{y}_{t_0},\mathbf{u}_{t_0},\mathbf{o}_{t_0},t_0)||p(\mathbf{z})).
\end{aligned}
$$

Plugging in the normalizing flows $\mathbf{a}^{(l)}$ for the posterior of $\mathbf{z}$,

$$\begin{aligned}\mathcal{L}(\boldsymbol{\phi}) = \mathbb{E}\Bigg[& \sum_k \mathbf{o}_{t_k} \odot \log \mathcal{N}(\mathbf{y}_{t_k}|\mathbf{x}(t_k), \boldsymbol{\sigma}_{\mathbf{y}}) + (1-c)\big[\log \lambda(a|\mathbf{x}(t), \mathbf{u}_{t_0}, t_0) + \log S(a|\mathbf{x}(t), \mathbf{u}_{t_0}, t_0)\big] \\ & + \int_{t_0}^{a} c \log S(t|\mathbf{x}(t), \mathbf{u}_{t_0}, t_0) dt + \int_{a}^{a_{\max}} (1-c) \log\left(1 - S(t|\mathbf{x}(t), \mathbf{u}_{t_0}, t_0)\right) dt \\ & - \frac{1}{2}\int_{t_0}^{a} \Big|\Big|\boldsymbol{\sigma}_x^{-1} \odot \big(\mathbf{W}\mathbf{x} + \mathbf{f}(\mathbf{x}(t), \mathbf{u}_{t_0}, t) - \mathbf{g}(\mathbf{x}(t), \mathbf{u}_{t_0}, t)\big)\Big|\Big|^2 dt\Bigg] \\ & - KL(q(\boldsymbol{\theta})||p(\boldsymbol{\theta})) - KL(q(\mathbf{z}^{(0)}|\mathbf{y}_{t_0}, \mathbf{u}_{t_0}, \mathbf{o}_{t_0}, t_0)||p(\mathbf{z}^{(0)})) + \sum_{l=1}^{L} \log\left|\det \frac{\partial a^{(l)}(\mathbf{z}^{(l)}, \boldsymbol{\gamma}_z, \phi_z)}{\partial \mathbf{z}^{(l)}}\right|.\end{aligned}$$

Here we do not show the variational parameters $\boldsymbol{\phi}$ in the notation for the approximate posteriors q and the parameters $\boldsymbol{\theta}$ from the conditional distributions for simplicity. Additionally, we have averaged over the imputed or generated $\mathbf{x}_0$. This is the objective function used in the methods.

II. NON-RECURRENT NEURAL NETWORK MORTALITY RATE

In our network model presented in the main results, we model the mortality rate with a recurrent neural network (RNN). This allows the use of a history of health to compute the mortality rate. We have also tested a model where we instead use a feed-forward neural network taking $\mathbf{x}(t), \mathbf{u}_{t_0}, t$ as input – this allows no memory of previous states to determine mortality. We use the same layer sizes as the recurrent neural network model, and use ELU activations.

III. GENERATED SYNTHETIC POPULATION

We have made a synthetic population available at `https://zenodo.org/record/4733386`. This population includes 3 million individuals for each baseline age of 65, 75, and 85 years old, for a total of 9 million individuals. The background health state has been generated by sampling based on the age and sex-dependent ELSA population. For binary variables we sample a 0 or 1 based on the observed sex and age-dependent prevalence, for continuous variables we sample from a Gaussian distribution with mean and standard deviation from the observed sex and age-dependent ELSA training sample mean and standard deviation. We set all individuals with no medications.

Using this input, we sample a baseline state for each synthetic individual and simulate their health trajectories for 20 years.

TABLE A. Variables used from the ELSA dataset. Background variables are only used at the first time-step, as $\mathbf{u}_{t_0}$. Longitudinal variables are predicted in $\mathbf{y}_t$. All variables are z-scored; additional transformations before z-scoring are indicated.

**Variable**	**Category**	**Wave type**	**Transformation**
Gait speed (average of 3 measurements, speed over 8 feet, age 60+)	Longitudinal	Self-report	
Dominant hand grip strength (average of 3 measurements)	Longitudinal	Nurse	
Non-dominant hand grip strength (average of 3 measurements)	Longitudinal	Nurse	
ADL score (count from 0-10, see Table B)	Longitudinal	Self-report	
IADL score (count from 0-13, see Table B)	Longitudinal	Self-report	
Time to rise from a chair 5x	Longitudinal	Nurse	
Time held leg raise (eyes open, maximum 30 secs)	Longitudinal	Nurse	Log-scaled
Time held full tandem stance (maximum 30 secs)	Longitudinal	Nurse	Log-scaled
Self-rated health (scored 0=excellent to 1=poor, 5 levels)	Longitudinal	Self-report	
Eyesight (with aids) (scored 0=excellent 1=legally blind, levels=6)	Longitudinal	Self-report	
Hearing (with aids) (scored 0=excellent to 1=poor, 5 levels)	Longitudinal	Self-report	
Walking ability score (unaided ability to walk 1/4 mile) (scored 0=no difficulty to 1=unable to do this, 4 levels)	Longitudinal	Self-report	
Diastolic blood pressure (average of 3 measurements)	Longitudinal	Nurse	
Systolic blood pressure (average of 3 measurements)	Longitudinal	Nurse	
Pulse (average of 3 measurements)	Longitudinal	Nurse	
Triglycerides	Longitudinal	Nurse	Log-scaled
C-reactive protein	Longitudinal	Nurse	Log-scaled
HDL cholesterol	Longitudinal	Nurse	
LDL cholesterol	Longitudinal	Nurse	
Glucose (fasting)	Longitudinal	Nurse	
Insulin-like growth factor 1	Longitudinal	Nurse	
Hemoglobin	Longitudinal	Nurse	
Fibrinogen	Longitudinal	Nurse	
Ferritin	Longitudinal	Nurse	Log-scaled
Total cholesterol	Longitudinal	Nurse	
White blood cell count	Longitudinal	Nurse	Log-scaled
Mean corpuscular haemoglobin	Longitudinal	Nurse	Log-scaled
Glycated hemoglobin (HgbA1c) (%)	Longitudinal	Nurse	
Vitamin-D	Longitudinal	Nurse	Log-scaled
Long-standing illness (yes/no)	Background	Self-report	
Long-standing illness limits activities (yes/no)	Background	Self-report	
Everything is an effort lately (yes/no)	Background	Self-report	
Ever smoked (yes/no)	Background	Self-report	
Currently smoke (yes/no)	Background	Self-report	
Height	Background	Nurse	
Body mass index (weight/height^2)	Background	Nurse	
Mobility status (1=walking without help/support, 0=walking requires help/support, bed bound, wheelchair, uncertain impairment)	Background	Nurse	
Country of birth (UK/outside UK)	Background	Self-report	
Drink alcohol (last 12 months, scored 1=almost every day to 6=every couple of months)	Background	Self-report	
Ever had a joint replacement (yes/no)	Background	Self-report	
Ever had bone fractures (yes/no)	Background	Self-report	
Sex	Background	Self-report	
Ethnicity (white/non-white)	Background	Self-report	
Hypertension medication (yes/no)	Background	Self-report	
Anticoagulant medication (yes/no)	Background	Self-report	
Cholesterol medication (yes/no)	Background	Self-report	
Hip/knee treatment (medication or exercise, yes/no)	Background	Self-report	
Lung/asthma medication (yes/no)	Background	Self-report	

TABLE B. Activities of daily living (ADL) and Instrumental activities of daily living (IADL) from the ELSA dataset, for a total of 10 ADL and 13 IADL.

**Activities of daily living (ADL)**
Walking 100 yards
Sitting for about two hours
Getting up from a chair after sitting for long periods
Climbing several flights of stairs without resting
Climbing one flight of stairs without resting
Stooping, kneeling, or crouching
Reaching or extending arms above shoulder level
Pulling/pushing large objects like a living room chair
Lifting/carrying over 10 lbs, like a heavy bag of groceries
Picking up a 5p coin from a table

**Instrumental activities of daily living (IADL)**
Dressing, including putting on shoes and socks
Walking across a room
Bathing or showering
Eating, such as cutting up your food
Getting in or out of bed
Using the toilet, including getting up or down
Using a map to get around a strange place
Preparing a hot meal
Shopping for groceries
Making telephone calls
Taking medications
Doing work around the house or garden
Managing money, eg paying bills and keeping track of expenses

TABLE C. Neural network architectures used in the DJIN model, as described in Fig 1 and "Network architecture and Hyperparameters" of the methods. The health variables $\mathbf{y}_{t_0}$ are size $N = 29$, the health variable observed mask $\mathbf{o}_{t_0}$ is of size $N = 29$, and the background health variables $\mathbf{u}_{t_0}$ with appended missing mask are of size $B + 17 = 36$.

**Encoder (VAE)**	
Layer #	Description
1	Input $(\mathbf{y}_{t_0}, \mathbf{o}_{t_0}, \mathbf{u}_{t_0}, t_0)$
2	(2N+B+17+1)x95 Fully connected layer
3	Batchnorm
4	ELU
5	95x70 Fully connected layer
6	Batchnorm
7	ELU
8	70x50 Fully connected layer

**Decoder (VAE)**	
Layer #	Description
1	Input $(\mathbf{z}, \mathbf{u}_{t_0}, t_0)$
2	(20+B+17+1)x65 Fully connected layer
3	Batchnorm
4	ELU
5	65xN Fully connected layer

**Diagonal dynamics f_i**	
Layer #	Layer description
1	Input $(x_i(t), t, \mathbf{u}_{t_0})$
2	(2+B+17)x12 Fully connected layer
3	ELU
4	12x1 Fully connected layer

**Mortality rate λ**	
Layer #	Layer description
1	Input $(\mathbf{x}(t), t)$
2	(N+1)x25 GRU
3	25x10 GRU
4	ELU
5	10x1 Linear layer

**Posterior drift function g**	
Layer #	Layer description
1	Input $(\mathbf{x}(t), \mathbf{u}_{t_0}, t)$
2	(N+B+17+1)x8 Fully connected layer
3	ELU
4	8xN Fully connected layer

**Inferring $\mathbf{h}_{t_0}$**	
Layer #	Layer description
1	Input $(\mathbf{x}(t_0), \mathbf{u}_{t_0}, t_0)$
2	(N+B+17+1)x75 Fully connected layer
3	ELU
4	75x40 Fully connected layer

**Normalizing flow a**	
Layer #	Layer description
1	Input $(\mathbf{z}^{(0)}, \boldsymbol{\gamma})$
2	30x24 Fully connected layer
3	BatchNorm
4	Tanh
5	24x20 Fully connected layer

**Dynamical noise strength $\boldsymbol{\sigma}_{\mathbf{x}}$**	
Layer #	Layer description
1	Input $(\mathbf{x}(t))$
2	NxN Fully connected layer
3	ELU
4	NxN Fully connected layer
5	Sigmoid

IV. SUPPLEMENTAL FIGURES

In S1 Fig we show the variables used in the ELSA data set, and the number of individuals for which each variable is observed at each year from the time of entrance to the study. The shaded fills indicate the proportion of observed variables (with respect to the maximum of that variable), with the darkest fill indicating almost 100%. Most variables are unobserved at any given time – which reinforces the need for effective baseline imputation. The full names of these variables are provided in supplemental Table A.

In S4 Fig, we show the relative RMSE of our model predictions and the elastic net linear model predictions for each health variable between 1 and 6 years – plotted against the proportion of observations for which the variable is missing in the full dataset. Our model predictions are generally worse for the variables with a higher proportion missing, with observable degradation for proportions of missing around 0.95 where accuracy goes above the sample mean predictions, although our model is always better than the elastic net linear model.

In S5 Fig we show 3 different example individuals from the test set and the model predicted trajectories. We choose the 6 of the best predicted variables to show. These predictions show the estimated uncertainty for these individual trajectories, and the variety in behaviour in the data for different individuals. The relative RMSE for these individuals averaged over each time point is shown, for comparison with Fig 2 in the main results.

In S11 Fig we show the generated synthetic population Kaplan-Meier survival curve (red line and shading) and the observed population Kaplan-Meier survival curve (blue line and shading) with 95% confidence intervals indicated by the shading. The same censoring distribution seen in the observed sample is applied to the synthetic population by sampling censoring ages above the baseline age from the test data with replacement. Agreement is good until ~ 90 years, after which the number of individuals observed in the dataset is very low.

In S8 Fig we show the classification accuracy for a logistic regression model discriminating between the synthetic and observed samples. Our model generated a synthetic population that is almost indistinguishable from the observed sample for most individuals, only rising to 60% accuracy at 18 years from baseline.

In S9 Fig we show the one-dimensional marginal distributions for each health variable for the generated synthetic population and observed sample at baseline. We see the synthetic population agrees with the observed sample, but often has a slightly lower variance. In S10 Fig we show the mean and standard deviation of generated synthetic population trajectories (red lines and shading) and the observed sample trajectories (blue lines and shading). The synthetic trajectories have somewhat lower variance but reasonable agreement with the means.

In S14 Fig, we contrast our network model's weight matrix with a pair-wise Pearson correlation network, where weights have been pruned with a p-value above 0.01 to match the 99% credible intervals used in our approach. We see many differences. Our weight matrix is much more sparse, including only the links useful for prediction. Our network is also directed and asymmetric, and one-way links between variables are observed, as well as distinct strengths of links in the different directions. However, the sign of the links in the weight matrix is typically the same as in the correlation network.

In S12 Fig we show, for each network weight, the posterior mean of the weight vs. the proportion of the approximate posterior distribution that is above zero for posterior weights, or below zero for negative weights. We exclude weights when the probability of the weight being in the opposite direction of the mean is above 1%. This approach only accepts connections with a large probability of having a definite sign. We see that large weights only have a small proportion of the posterior with the opposite sign; showing that the strong connections inferred by the model are robust.

Several alternative models were explored, as described in the Latent Variable Models section of the Methods and Supplemental Sec. II. In S7 Fig we summarize predictions for the one-dimensional summary model, in which dynamics are built on one latent summary health variable. This model performs worse than our DJIN model for both health and survival, and is often even worse than a static baseline prediction model (blue squares) for health. In S6 Fig we show model results with a full neural network drift function that includes all interactions for a 30-dimensional latent variable model, in contrast to the linear pair-wise network in our main results with the DJIN model. This shows that the full NN model only does slightly better than the pair-wise network model for health, and is slightly worse for survival. This indicates that the pair-wise network assumptions made by our DJIN model do not sacrifice much accuracy. In S2 Fig we show the model results with a feed-forward neural network for the mortality rate instead of a recurrent neural network (GRU). Our recurrent neural network (RNN) model achieves slightly better C-index and Brier scores, particularly for older ages. The models are nearly equivalent for longitudinal prediction.

In S3 Fig we show the D-calibration histogram comparison between the DJIN model and the elastic net Cox model. The histograms reflect the χ^2 and p-values given in the main results, showing that the both models have calibrated probabilities.
