# Supplementary figures and images for: Interpretable machine learning for high-dimensional trajectories of aging health
Source: PLoS Comput Biol. 2022 Jan 10;18(1):e1009746. doi: 10.1371/journal.pcbi.1009746 (PMC8782527; doi:10.1371/journal.pcbi.1009746)

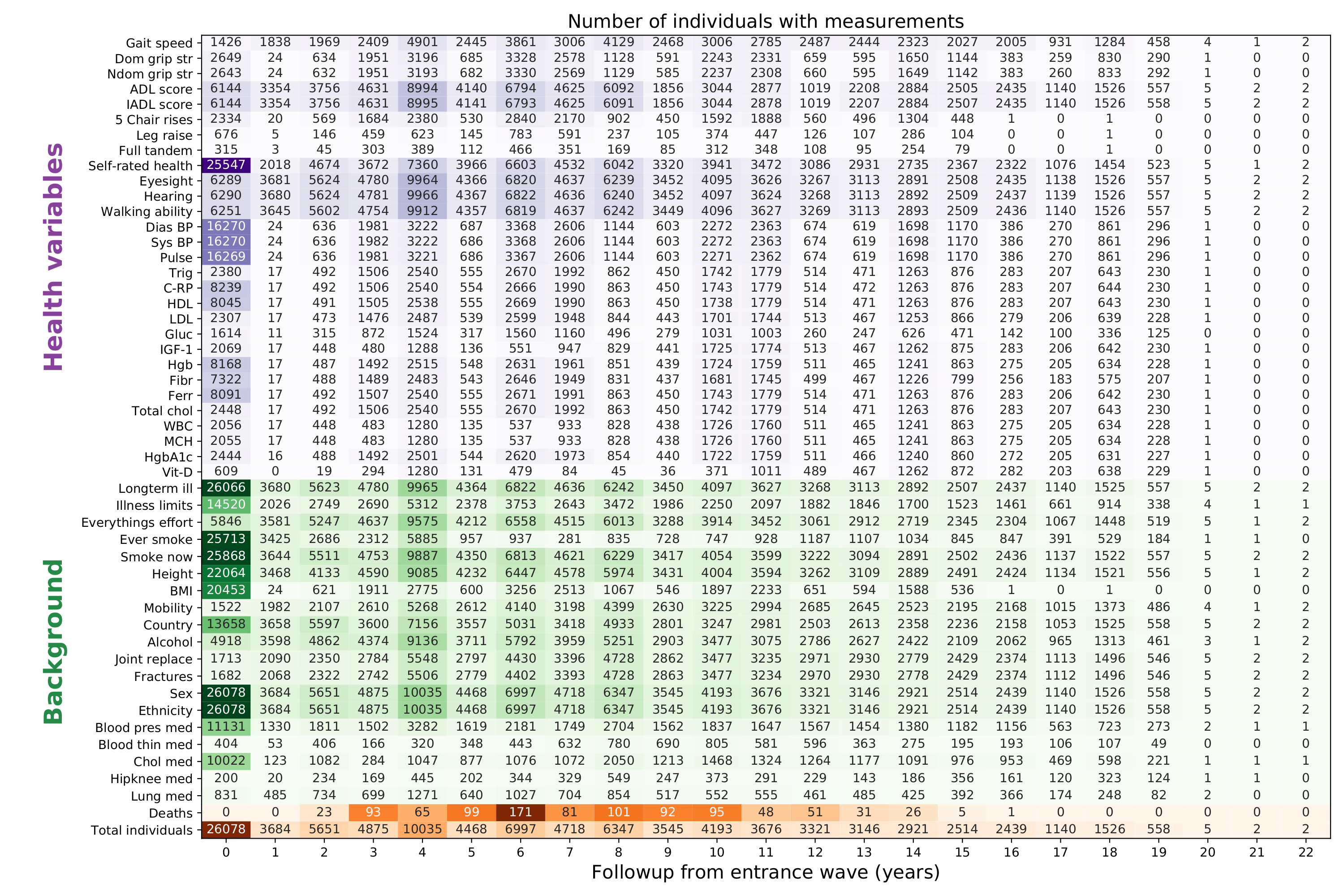

Supplement: S1 Fig — Number of individuals with measurements vs number of years after entrance to the study. Although ELSA study design has each wave 2 years apart, the age of individuals can change between 1 and 3 years between visits. Health variables (purple shading) are included in yt. Background variables (green shading) are included in ut0. Indicated at the bottom (orange shading) are the number of deaths reported, the number of individuals, and the average coverage percentage for those individuals. The darker shading indicates more measurements, relative to the maximum for that variable. (TIF) [file pcbi.1009746.s002.tif]

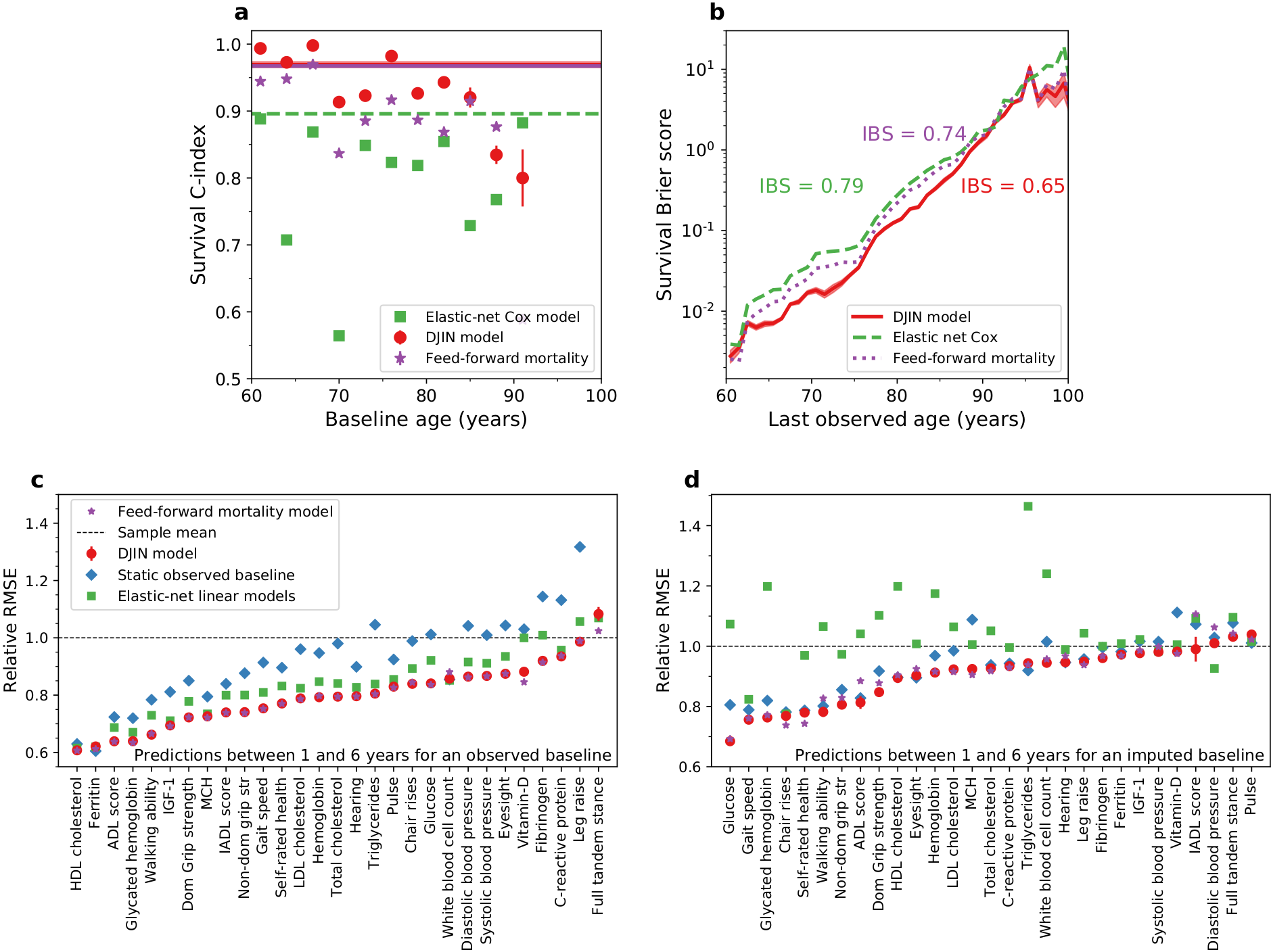

Supplement: S2 Fig — a) Time-dependent C-index stratified vs age (points) and for all ages (line). Results are shown for the feed-mortality mortality rate model (purple), the DJIN network model with a recurrent neural network mortality rate shown in the main results (red) and a Elastic net Cox model (green). (Higher scores are better). b) Brier scores for the survival function vs death age. Integrated Brier scores (IBS) over the full range of death ages is also shown. The Breslow estimator is used for the baseline hazard in the Cox model (Cox-Br). (Lower scores are better). Our DJIN model performs better than the feed-forward mortality model. c) RMSE scores when the baseline value is observed for each health variable for predictions at least 5 years from baseline, scaled by the RMSE score from the age and sex dependent sample mean (relative RMSE scores). We show the predictions from the feed-forward model starting from the baseline value (purple stars), our DJIN model (red circles), predictions assuming a static baseline value (blue diamonds), an elastic-net linear model (green squares). (Lower is better). d) Relative RMSE scores when the when the baseline value for each health variable is imputed for predictions past 5 years from baseline. We show the predictions from the feed-forward mortality model starting from the imputed value (purple stars), our DJIN model (red circles), and predictions with an elastic-net linear model (green squares). For longitudinal predictions, the DJIN model is almost equivalent to the feed-forward mortality model. (TIF) [file pcbi.1009746.s003.tif]

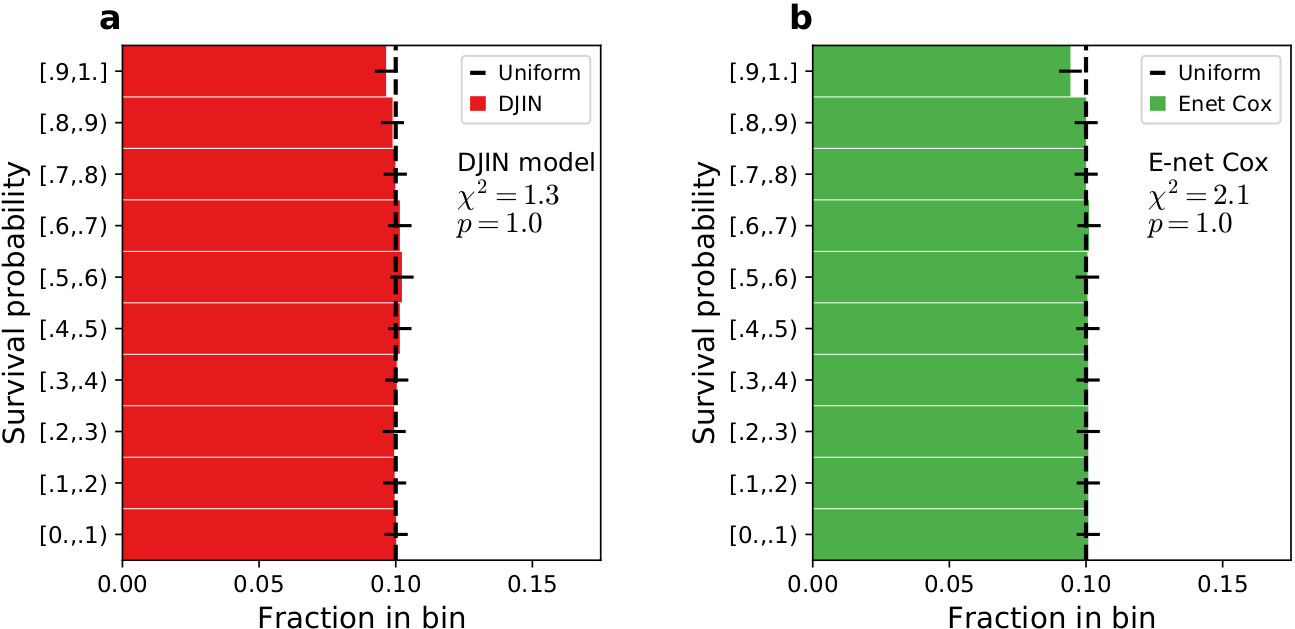

Supplement: S3 Fig — a) D-calibration of survival predictions for the DJIN model. Estimated survival probabilities are expected to be uniformly distributed (dashed black line). We use Pearson’s χ2 test to assess the distribution of survival probabilities finding χ2 = 1.3 and p = 1.0 and an elastic net Cox model. (Higher p-values and smaller χ2 statistics are better). b) D-calibration of survival predictions for the elastic-net Cox model. Estimated survival probabilities are expected to be uniformly distributed (dashed black line). We use Pearson’s χ2 test to assess the distribution of survival probabilities finding χ2 = 2.1 and p = 1.0. Error bars show the standard deviation. (TIF) [file pcbi.1009746.s004.tif]

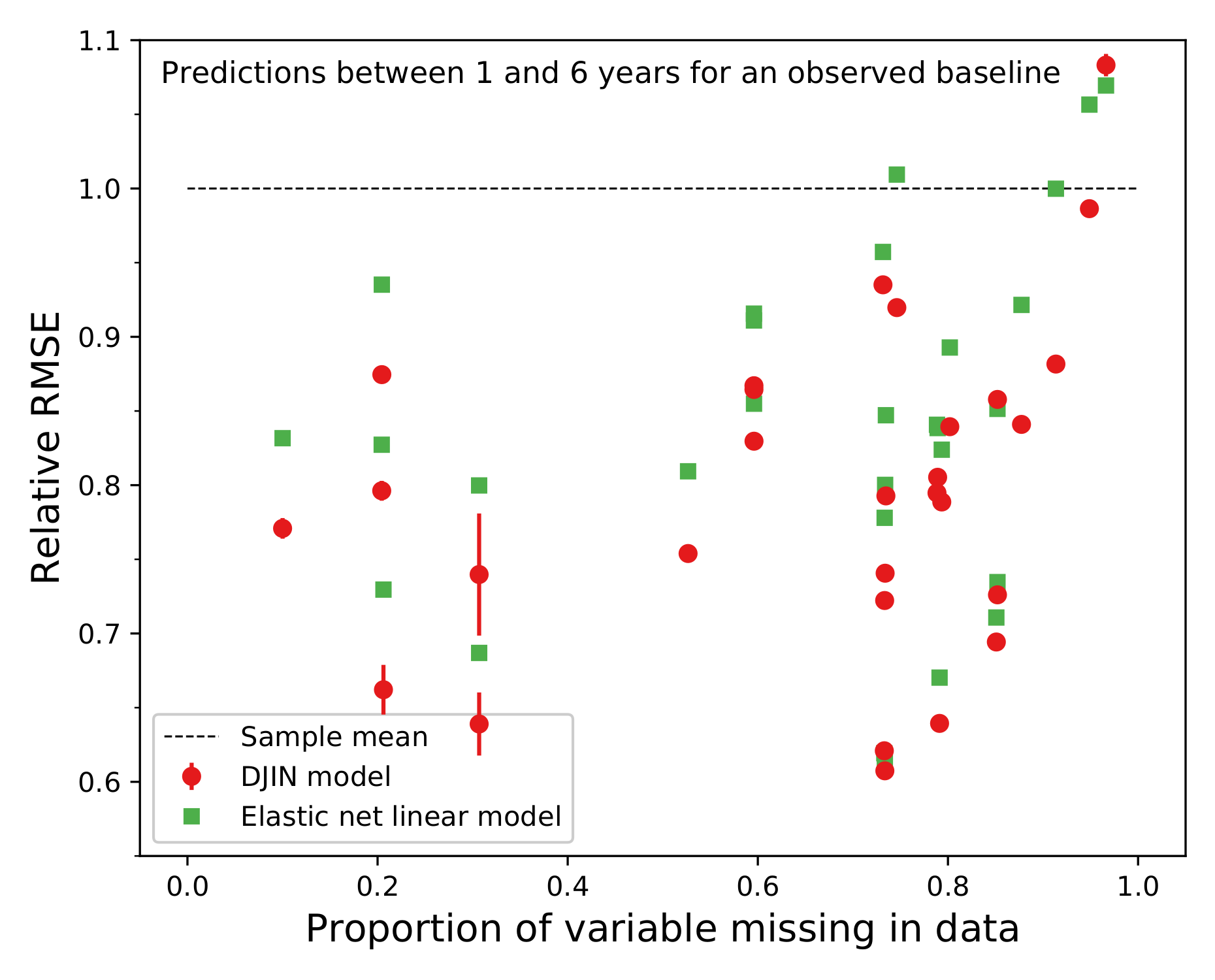

Supplement: S4 Fig — Relative RMSE up to six-years past baseline for longitudinal predictions of each health variable plotted against the proportion of observations where that variable was missing. Red circles show our network DJIN model, while green squares show the elastic net linear model. Predictions degrade only at high missingness. (TIF) [file pcbi.1009746.s005.tif]

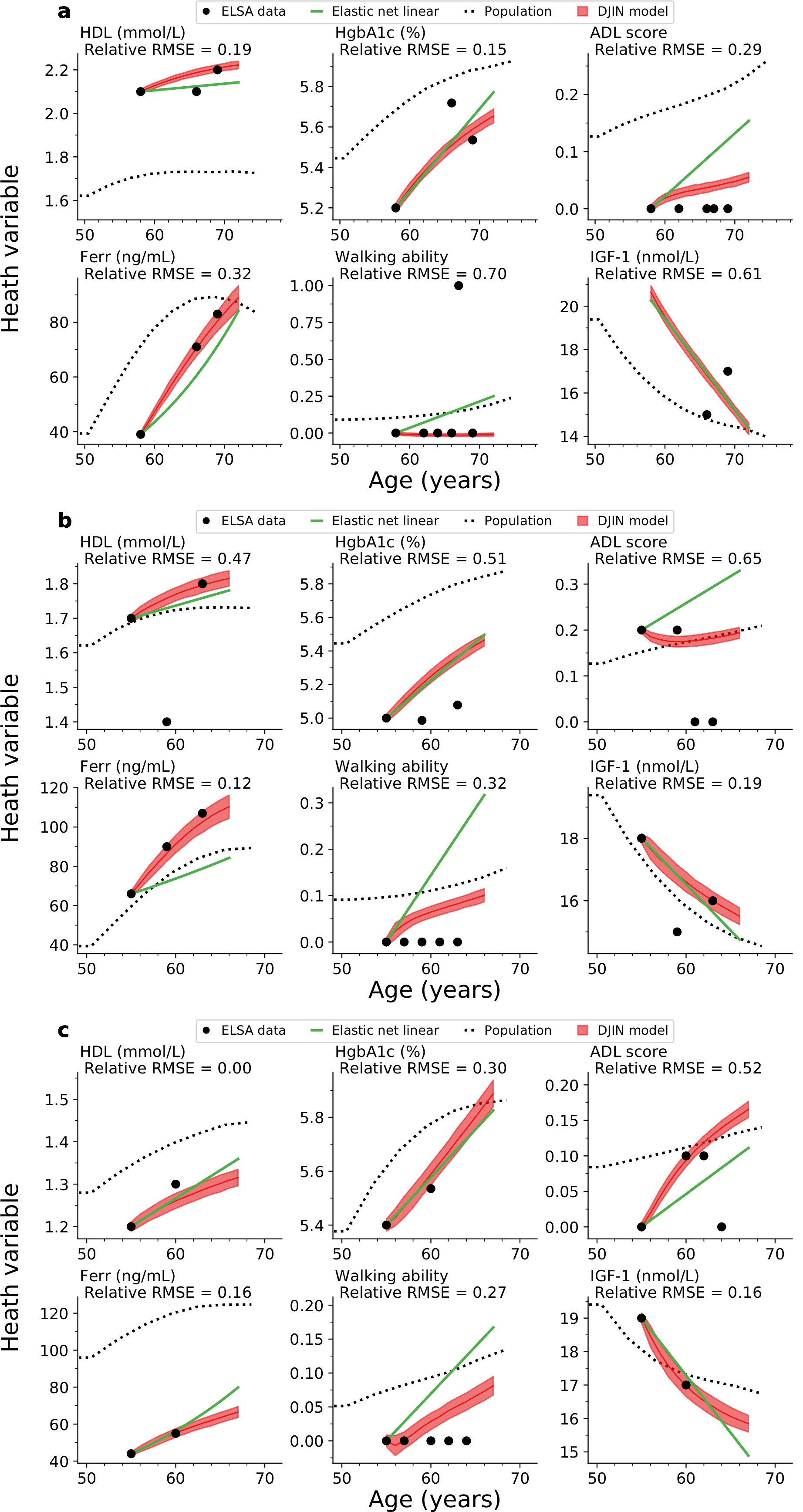

Supplement: S5 Fig — We show example predictions for 3 test individuals (a, b, and c). For each individual we show the top 6 best predicted health variables from Fig 2 in the main results. Black circles show the observed ELSA data. Red lines indicate the mean predicted x(t) and the red shaded region is one standard deviation from the predicted mean trajectory. Green lines indicate the linear model prediction (which appear curved for log-scaled variables such as ferritin). The average relative RMSE for each variable for each individual is shown. (TIF) [file pcbi.1009746.s006.tif]

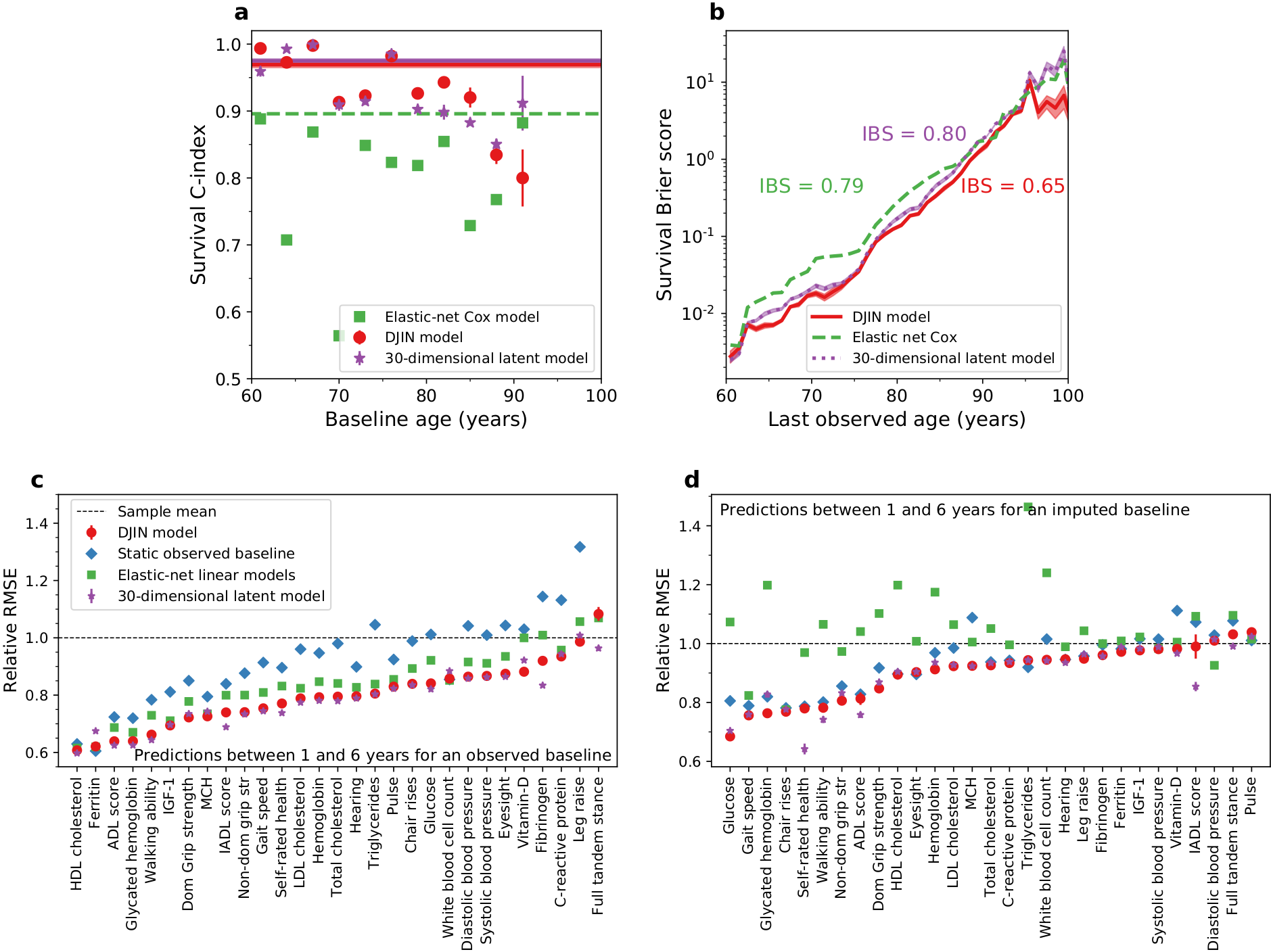

Supplement: S6 Fig — a) Time-dependent C-index stratified vs age (points) and for all ages (line). Results are shown for the full neural network model (purple), the DJIN network model shown in the main results (red) and a Elastic net Cox model (green). (Higher scores are better). b) Brier scores for the survival function vs death age. Integrated Brier scores (IBS) over the full range of death ages is also shown. The Breslow estimator is used for the baseline hazard in the Cox model (Cox-Br). (Lower scores are better). c) RMSE scores when the baseline value is observed for each health variable for predictions at least 5 years from baseline, scaled by the RMSE score from the age and sex dependent sample mean (relative RMSE scores). We show the predictions from the full neural network model starting from the baseline value (purple stars), our network model (red circles), predictions with a static baseline value (blue diamonds), an elastic-net linear model (green squares). (Lower is better). d) Relative RMSE scores when the when the baseline value for each health variable is imputed for predictions past 5 years from baseline. We show the predictions from the full neural network model starting from the imputed value (purple stars), our network model (red circles), and predictions with an elastic-net linear model (green squares). (TIF) [file pcbi.1009746.s007.tif]

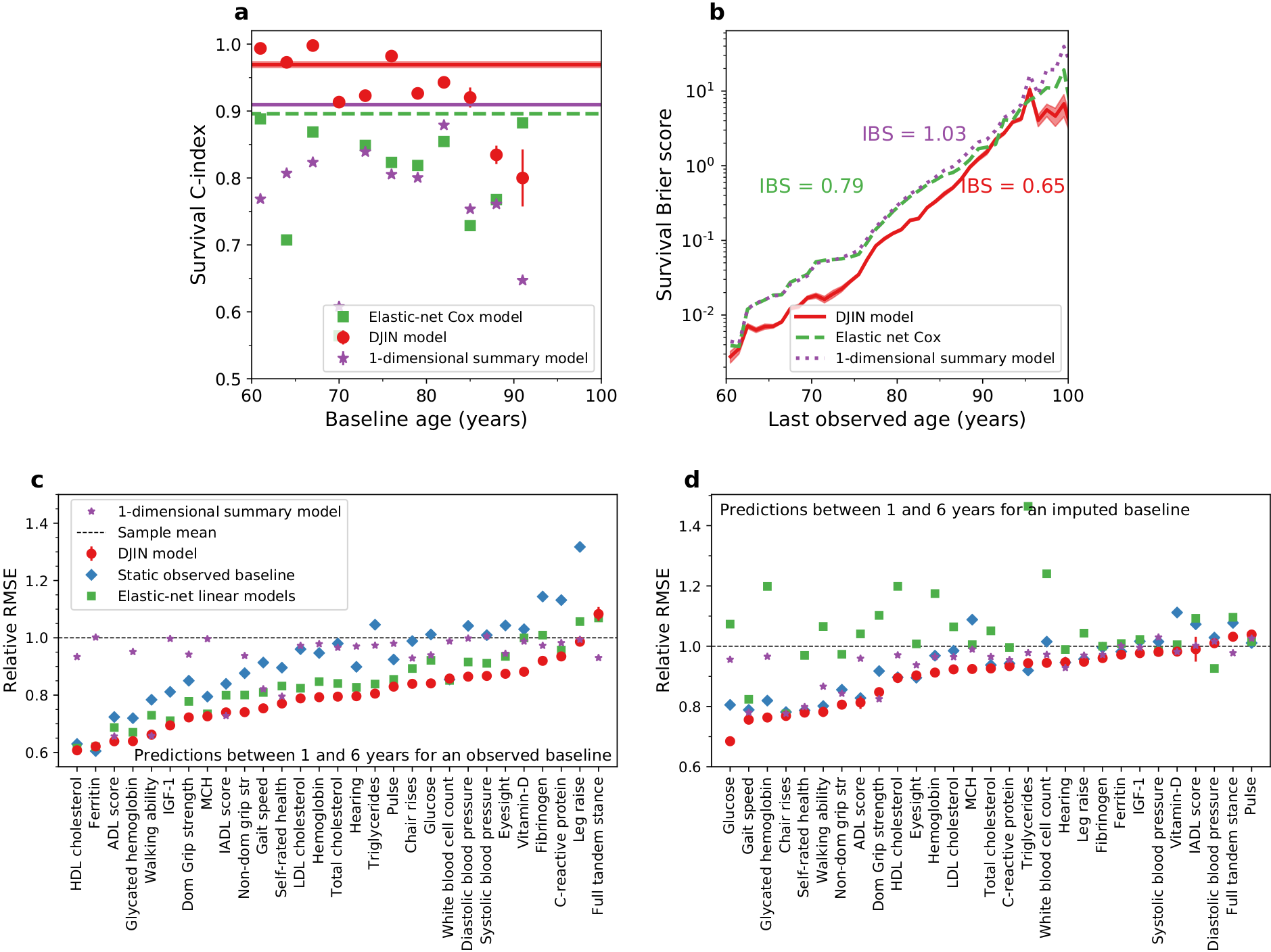

Supplement: S7 Fig — a) Time-dependent C-index stratified vs age (points) and for all ages (line). Results are shown for the 1D summary model (purple), the DJIN network model shown in the main results (red) and a Elastic net Cox model (green). (Higher scores are better). b) Brier scores for the survival function vs death age. Integrated Brier scores (IBS) over the full range of death ages is also shown. The Breslow estimator is used for the baseline hazard in the Cox model (Cox-Br). (Lower scores are better). c) RMSE scores when the baseline value is observed for each health variable for predictions at least 5 years from baseline, scaled by the RMSE score from the age and sex dependent sample mean (relative RMSE scores). We show the predictions from the 1D summary model starting from the baseline value (purple stars), our network model (red circles), predictions assuming a static baseline value (blue diamonds), an elastic-net linear model (green squares). (Lower is better). d) Relative RMSE scores when the when the baseline value for each health variable is imputed for predictions past 5 years from baseline. We show the predictions from the 1D summary model starting from the imputed value (purple stars), our network model (red circles), and predictions with an elastic-net linear model (green squares). (TIF) [file pcbi.1009746.s008.tif]

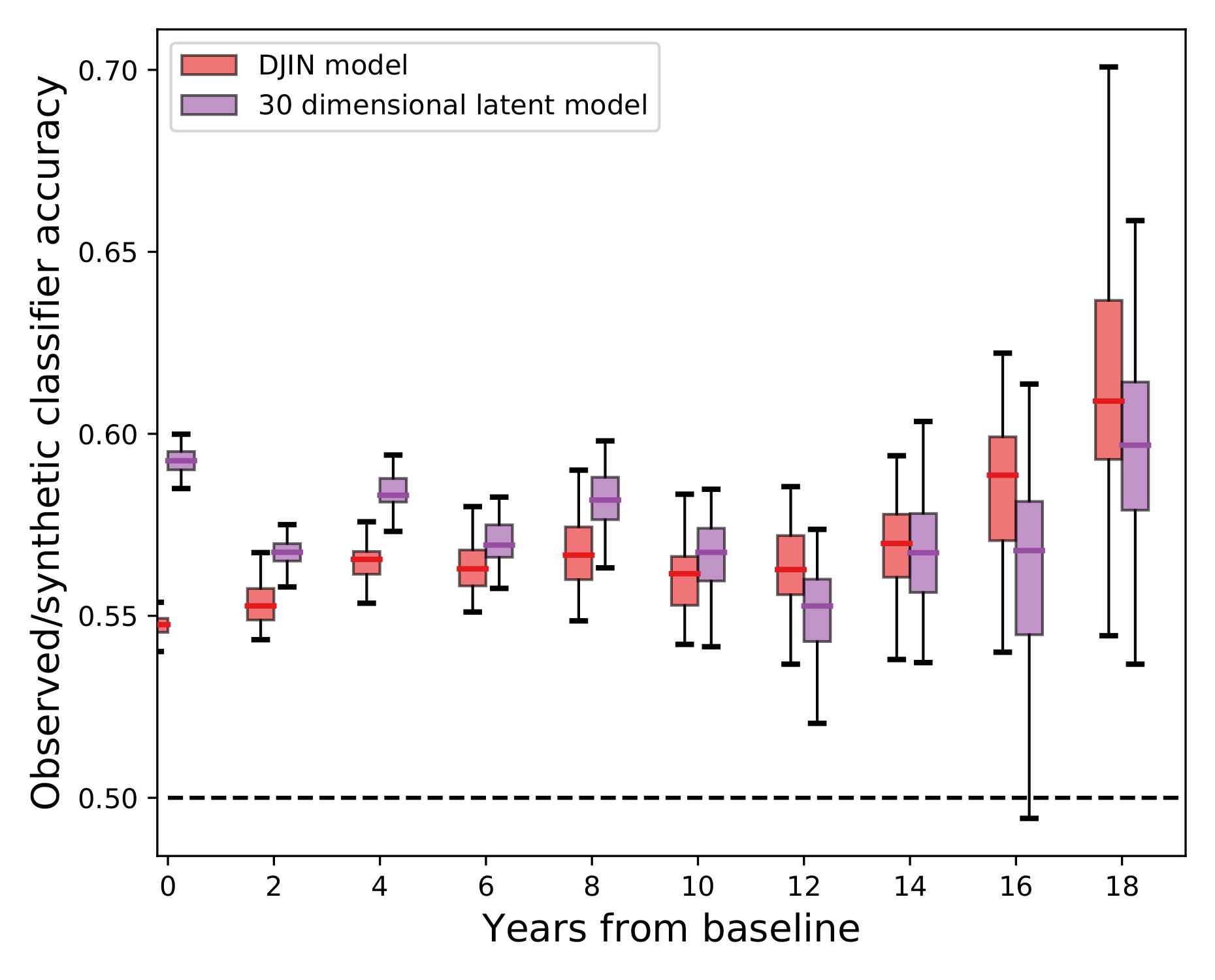

Supplement: S8 Fig — We use a logistic regression classifier to evaluate the quality of our generated synthetic population by the classifier’s ability to differentiate the synthetic population from the observed sample. The boxplot shows the median with the horizontal lines, interquartile range with the box, and 1.5x from the interquartile range with the whiskers. Completely indistinguishable natural and synthetic populations would have a classification accuracy of 0.5. We show the classification accuracy vs years from baseline, showing low classification accuracies that increase slowly with time from baseline in the DJIN model, and the DJIN model is equivalent or better than non-linear latent variable models. (TIF) [file pcbi.1009746.s009.tif]

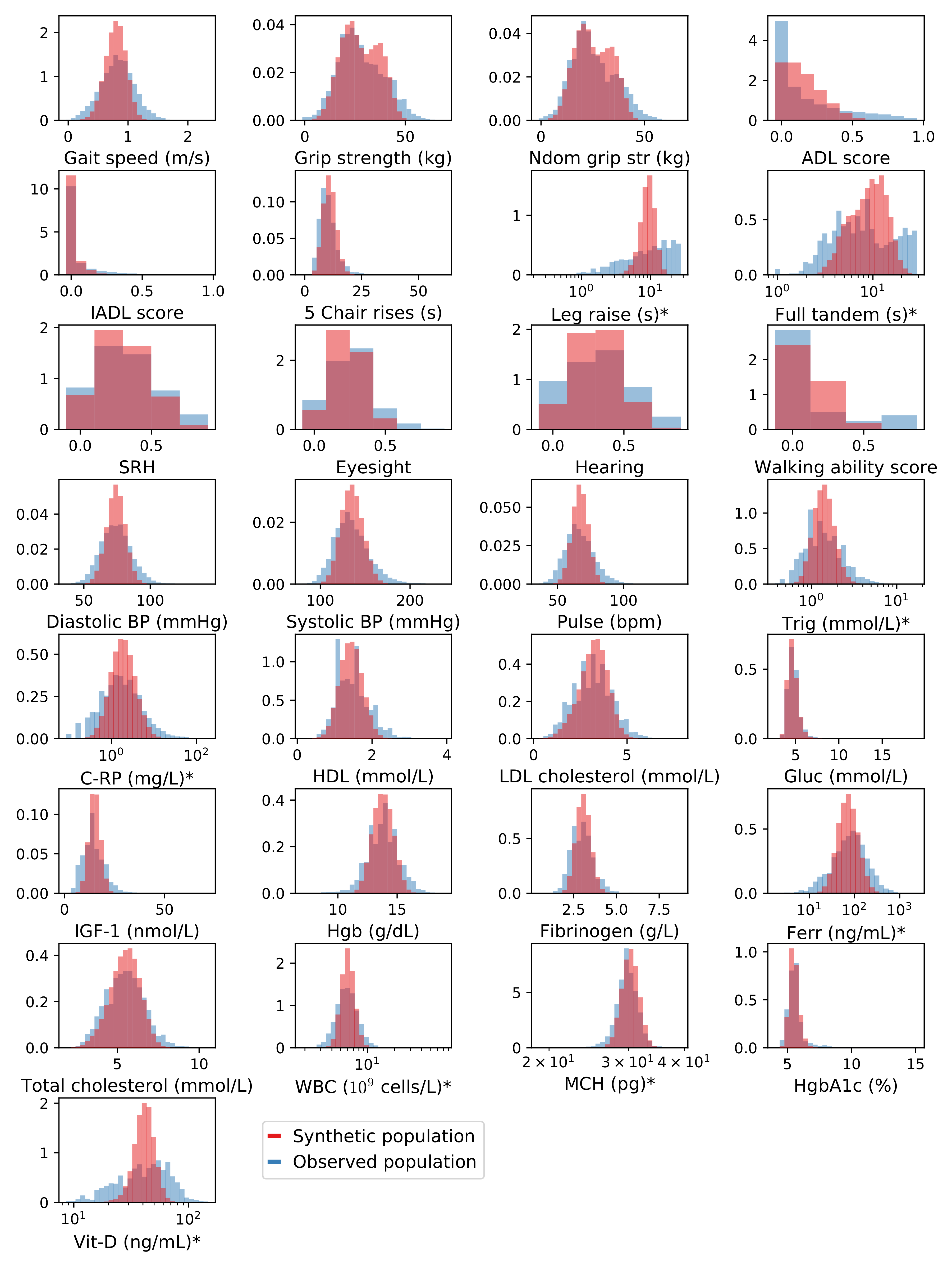

Supplement: S9 Fig — Each plot shows a synthetic baseline marginal distribution (red shading) for each variable. The synthetic baseline is generated given the background variables ut0 for the test set. Also shown is the observed distribution (blue shading). Log-scaled variables are shown with a logarithmic x-axis, and are indicated with an *. (TIF) [file pcbi.1009746.s010.tif]

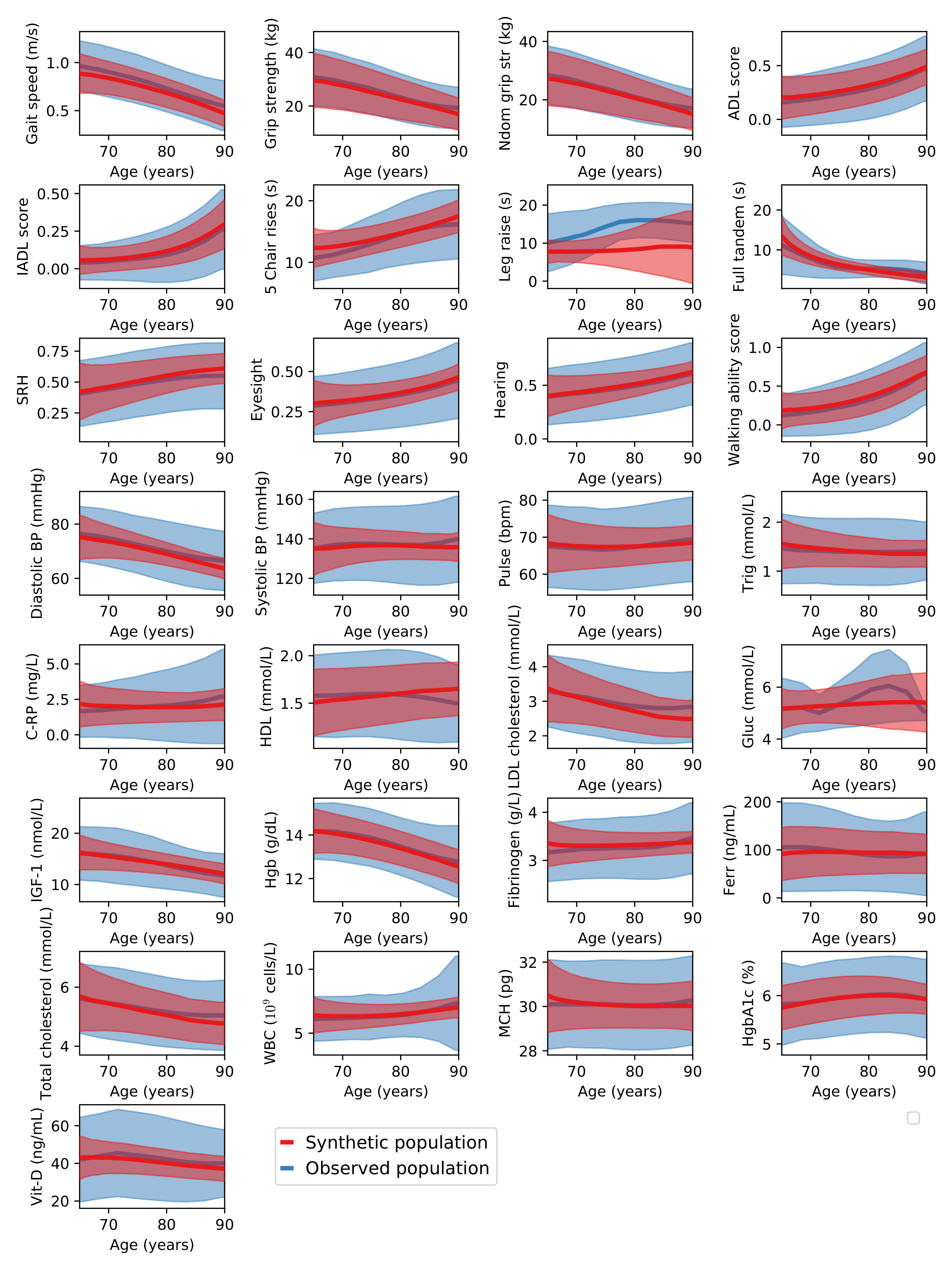

Supplement: S10 Fig — Red lines show the synthetic population trajectory marginal distribution means for each variable. Red shaded regions indicate 1 standard deviation away from the mean. Synthetic trajectories are generated from the baseline states shown in S9 Fig. Blue lines and shaded regions indicate the corresponding means and 1 standard deviation away for the observed population. (TIF) [file pcbi.1009746.s011.tif]

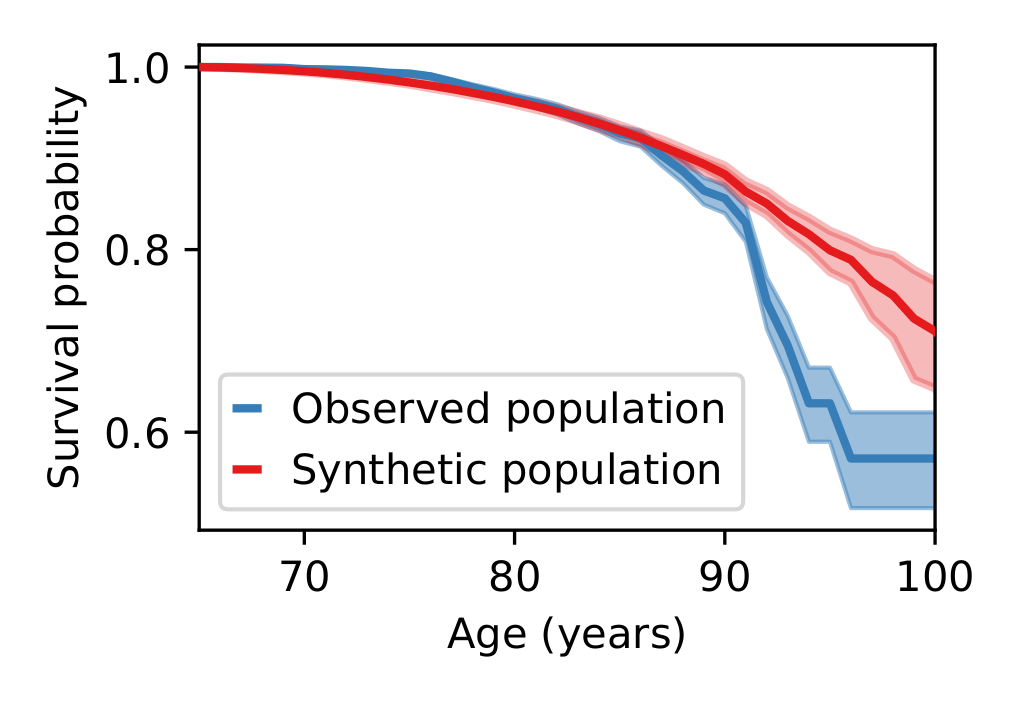

Supplement: S11 Fig — Survival curve for synthetic and observed populations, as indicated. The shaded regions show the 95% confidence intervals for Kaplan-Meier curves. The observed sample censoring distribution is applied to the synthetic population. The survival probability is approximately the same until 90 years, indicating that the mortality of the synthetic population is representative until older ages. (TIF) [file pcbi.1009746.s012.tif]

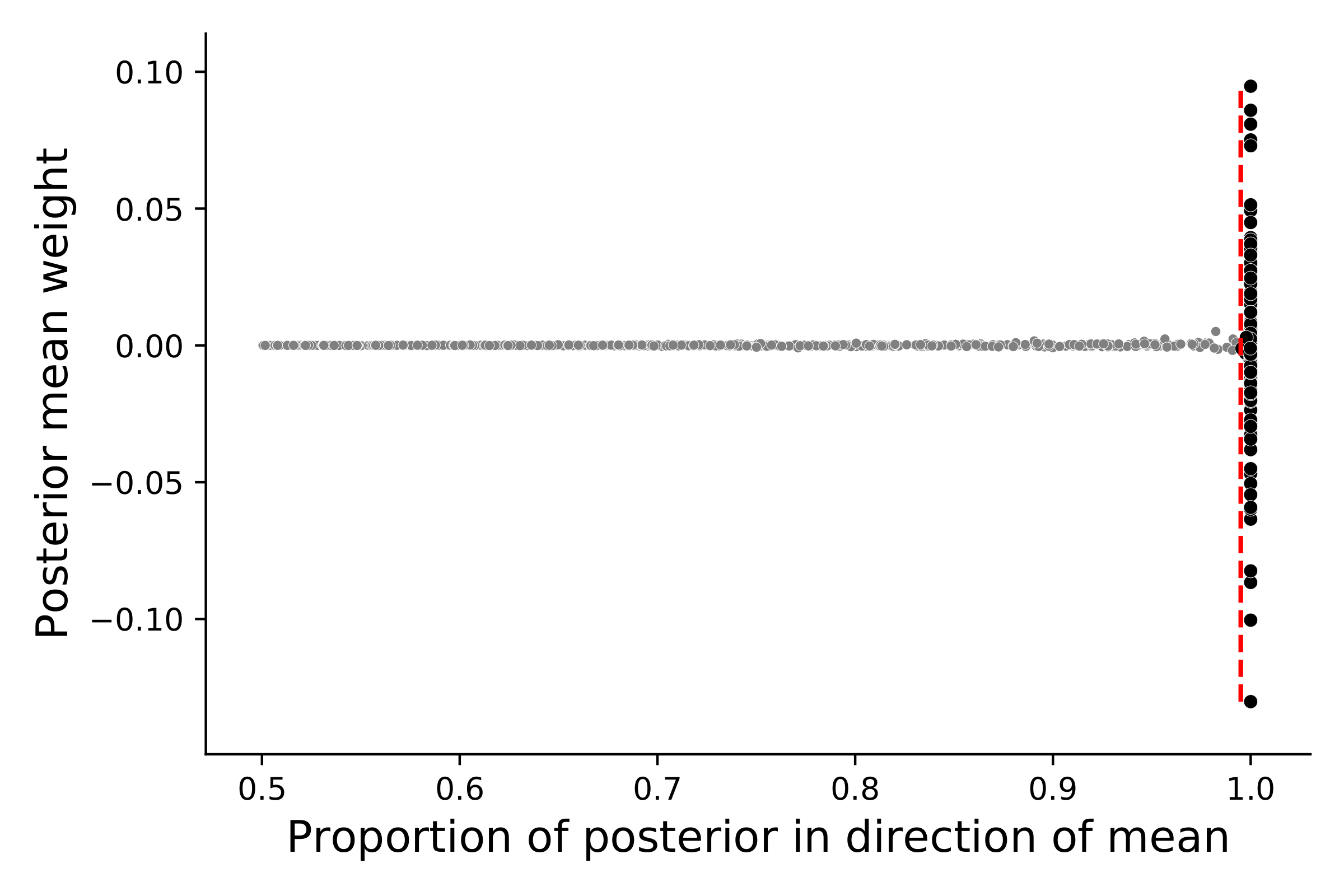

Supplement: S12 Fig — Criteria for determining robust connections. We show the posterior mean of the network weights {Wij} vs. the proportion of the posterior above zero for weights with a positive mean, and below zero for weights with a negative mean. The vertical dashed red line shows the criteria for robust connections (which are used in Fig 4), which is a 99% credible interval around the mean not containing zero. We see that larger weights are all credible, while many smaller weights are not. (TIF) [file pcbi.1009746.s013.tif]

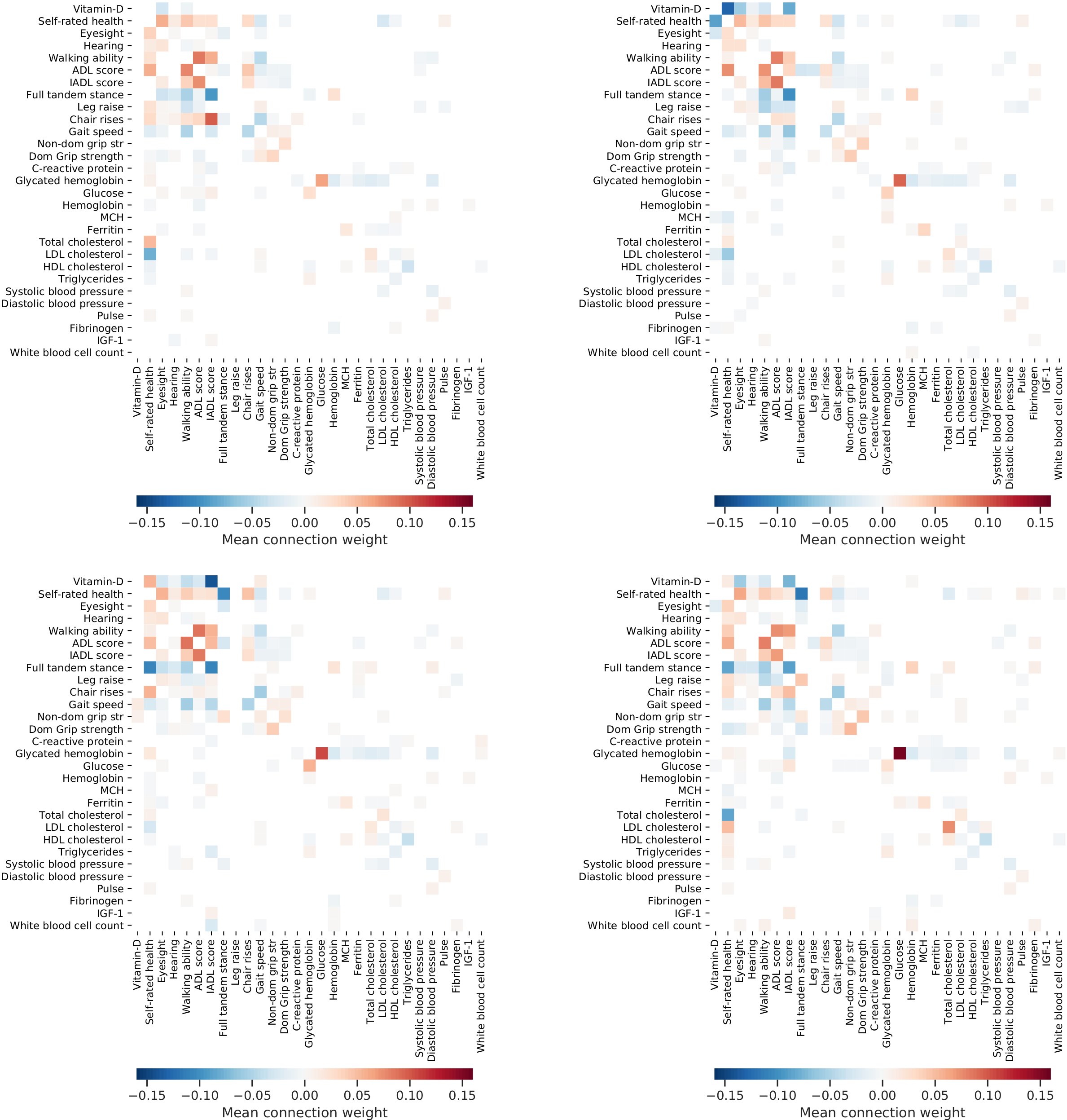

Supplement: S13 Fig — Inferred network for 4 different fits of the model. These networks visually look very similar, however there are some differences in magnitudes of the connections. (TIF) [file pcbi.1009746.s014.tif]

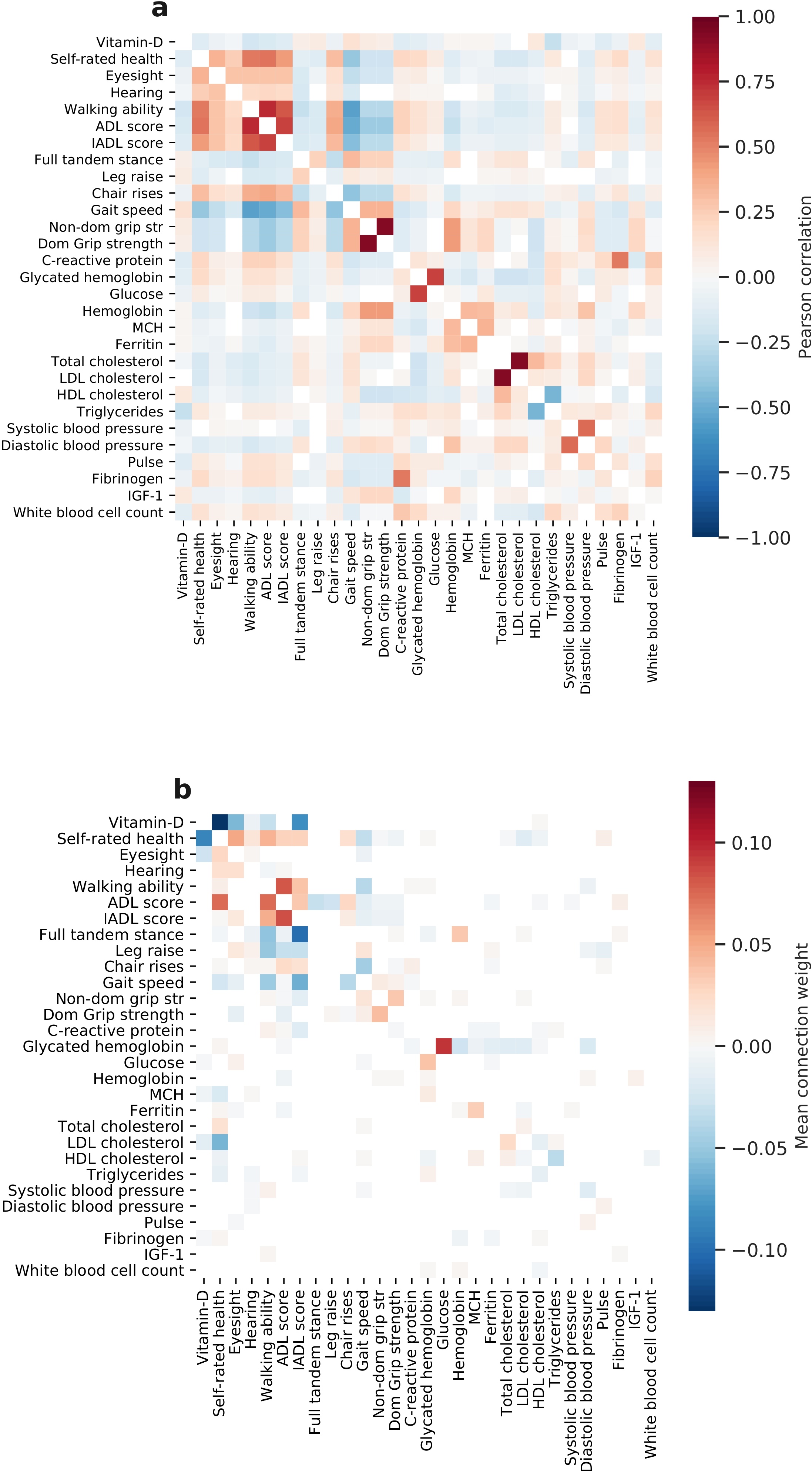

Supplement: S14 Fig — a) Pearson correlation network between the health variables for all individuals at all time-points, values are pruned for p-values above 0.01. b) Our model interaction network, for comparison. Weights are pruned when the 99% posterior credible interval includes zero. (TIF) [file pcbi.1009746.s015.tif]

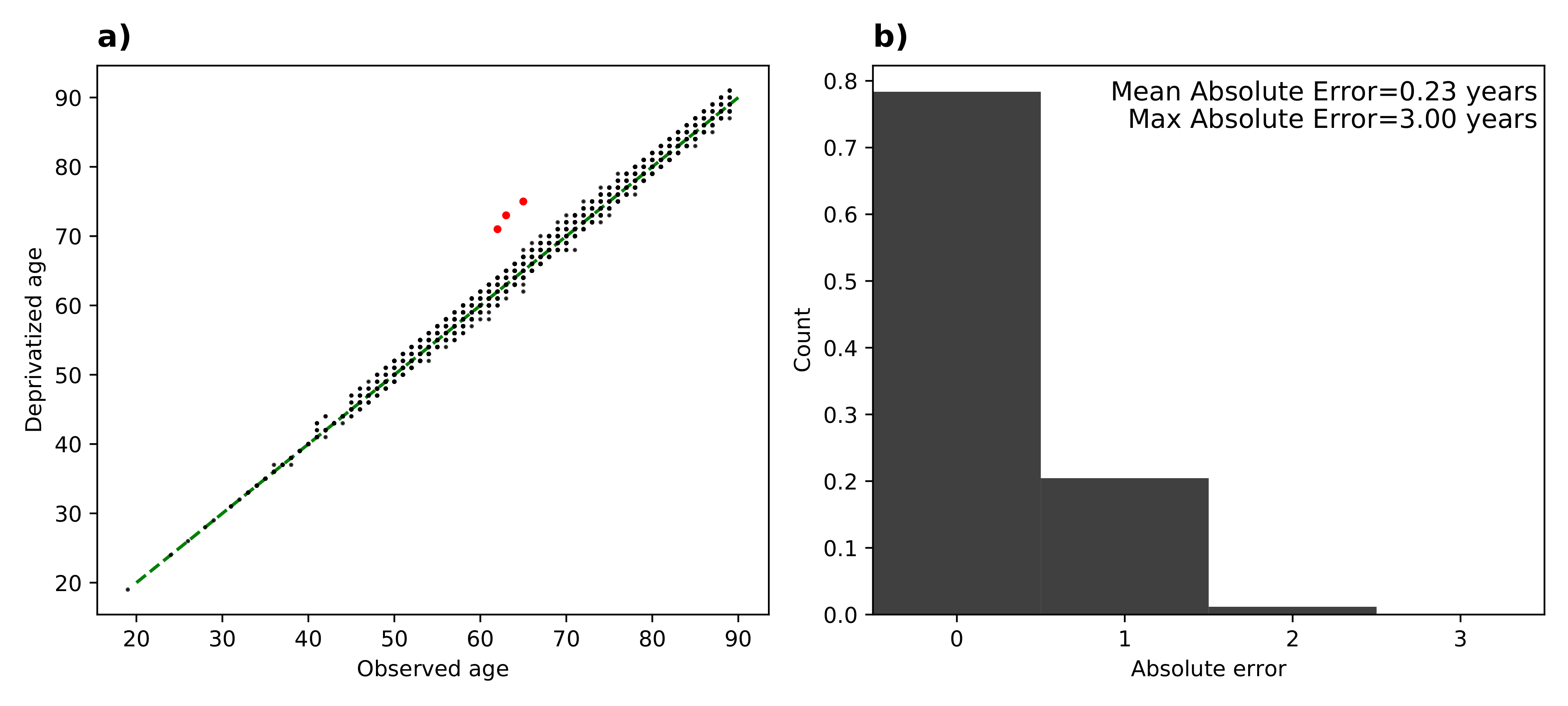

Supplement: S15 Fig — All known unprivatized ages past the first age for each individual in the ELSA dataset are set to missing. This allows us to test our approach to deprivatizing ages above 90, using the known ages below 90. a) Scatter plot of observed age vs deprivatized age. The green dashed light highlights perfect deprivatization. All errors are within 3 years, except for the three highlighted red points for 3 different individuals. For these individuals, we suspect a data error. For one of these individuals their observed age only advances 1 year over 5 waves, for another their age advances 15 years over 2 waves, and for the third their age advances 7 years over 1 wave. b) Dropping these three red points, we show that the mean absolute error with this approach is 0.23 years, and the maximum error is 3 years. The histogram shows that for the vast majority of individuals, there is a 0 to 1 year error with this method of deprivatization. (TIF) [file pcbi.1009746.s016.tif]

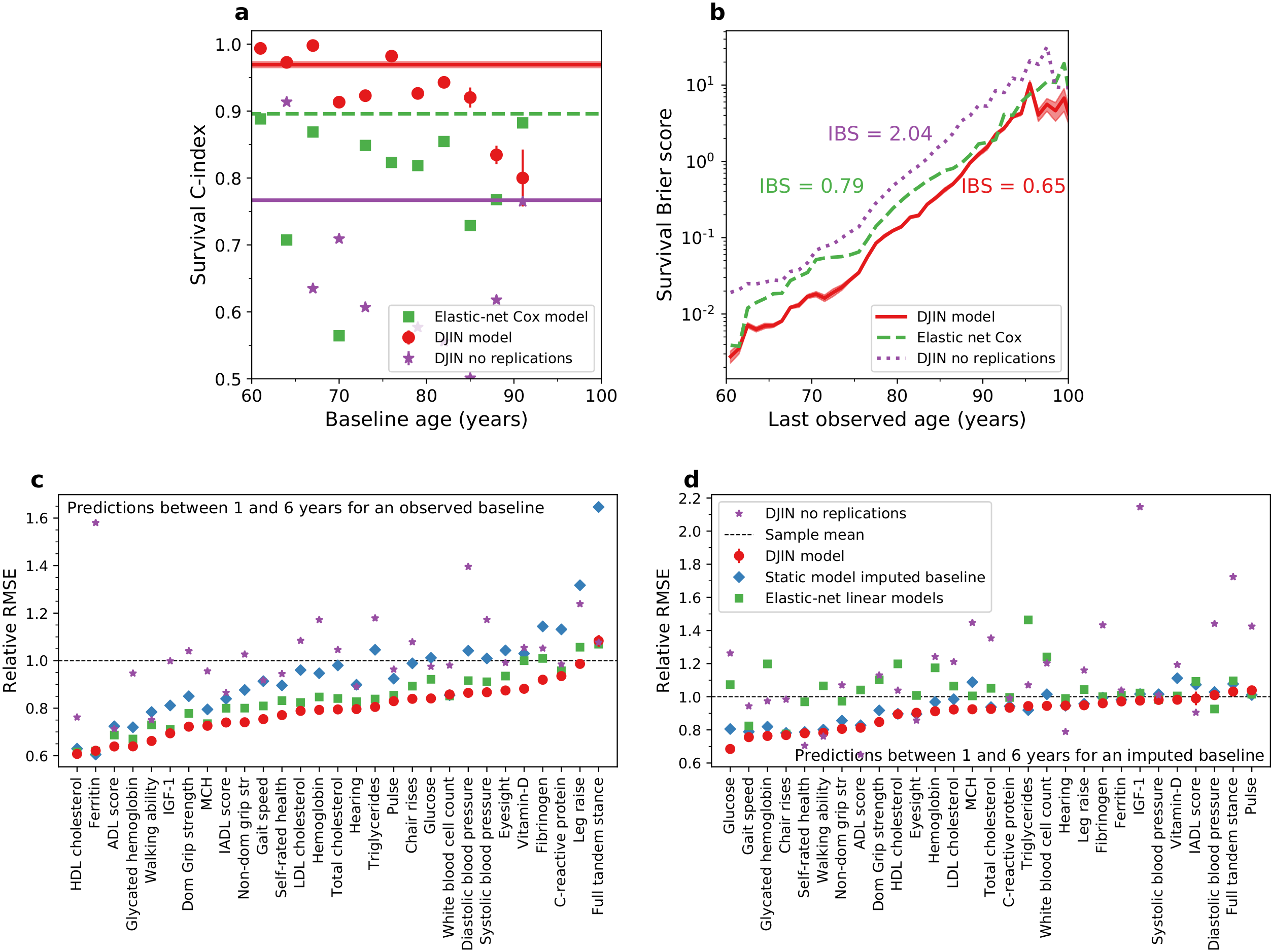

Supplement: S16 Fig — a) Time-dependent C-index stratified vs age (points) and for all ages (line). Results are shown for the DJIN model trained without replicated individuals (purple), the DJIN model trained with replicated individuals shown in the main results (red) and a Elastic net Cox model (green). (Higher scores are better). b) Brier scores for the survival function vs death age. Integrated Brier scores (IBS) over the full range of death ages is also shown. The Breslow estimator is used for the baseline hazard in the Cox model (Cox-Br). (Lower scores are better). c) RMSE scores when the baseline value is observed for each health variable for predictions at least 5 years from baseline, scaled by the RMSE score from the age and sex dependent sample mean (relative RMSE scores). We show the predictions from the DJIN model trained without replicated individuals starting from the baseline value (purple stars), the DJIN model trained with replicated individuals (red circles), predictions assuming a static baseline value (blue diamonds), an elastic-net linear model (green squares). (Lower is better). d) Relative RMSE scores when the when the baseline value for each health variable is imputed for predictions past 5 years from baseline. We show the predictions from the DJIN model trained without replicated individuals starting from the imputed value (purple stars), our DJIN model trained with replicated individuals (red circles), and predictions with an elastic-net linear model (green squares). (TIF) [file pcbi.1009746.s017.tif]
