# Supplementary material for: Interpretable machine learning for high-dimensional trajectories of aging health
Source: PLoS Comput Biol. 2022 Jan 10;18(1):e1009746. doi: 10.1371/journal.pcbi.1009746 (PMC8782527; doi:10.1371/journal.pcbi.1009746)
Supplement: Attachment — Submitted filename: djin response plos compbio.pdf [file pcbi.1009746.s018.pdf]

We thank the two reviewers for their comments and suggestions (italics below). We have addressed them in detail below, and in our revised draft. A significant addition to the draft is the new Fig 3, which explicitly examines the linear interactions and high-dimensionality of the DJIN model. Additionally, we have added three additional supplemental figures (see below).

<u>Reviewer 1:</u>

1. One part of description of the approach that is not entirely clear to me is section IV.B. In particular, if individuals were "replicated to use all possible starting points" then it seems (the combined likelihood for all individuals was not presented so it is just my (mis-?)interpretation) that the assumption that "replicated individuals are independent in the likelihood" is rather a strong one. If I get it right, this might also contribute to the observed underestimation of standard deviations. Could you please elaborate more on this? I wonder if it would be feasible to check if the approach without such replication of individuals leads to smaller underestimation of st. Dev.

> The combined likelihood for independent individuals in the data is the product of the likelihood for each individual (which is presented in the paper, the first 2 lines of Equation 18). However when we augment the data by replicating individuals to start at different baselines, these replicated individuals are not independent – so simply including them in the product amounts to an approximation.
>
> The effect of this approximation and other techniques used in deep learning such as batch-normalization (which we also use) on variational Bayesian inference has been studied (Wenzel et al. ICML 2020, appendix K). The result is that we are optimizing a smaller lower bound to the model evidence (Supplemental equation 1 and 2) than we otherwise would. This can result in a worse approximate posterior. Regardless of this, variational inference typically underestimates the width of the posterior. This is because minimizing the reverse KL-divergence between the approximate posterior q and posterior p is asymmetric and involves integrating over q, so distant parts of the distribution where the disagreement between q and p is greater can be effectively ignored when q is small.
>
> When we do not replicate individuals to augment our data, we obtain significantly worse health and survival predictions (new Supplemental Figure 16), and mean trajectories of the synthetic population that do not match the observed data. However, there are modest but noticeable improvements to standard deviations (shown on next page).

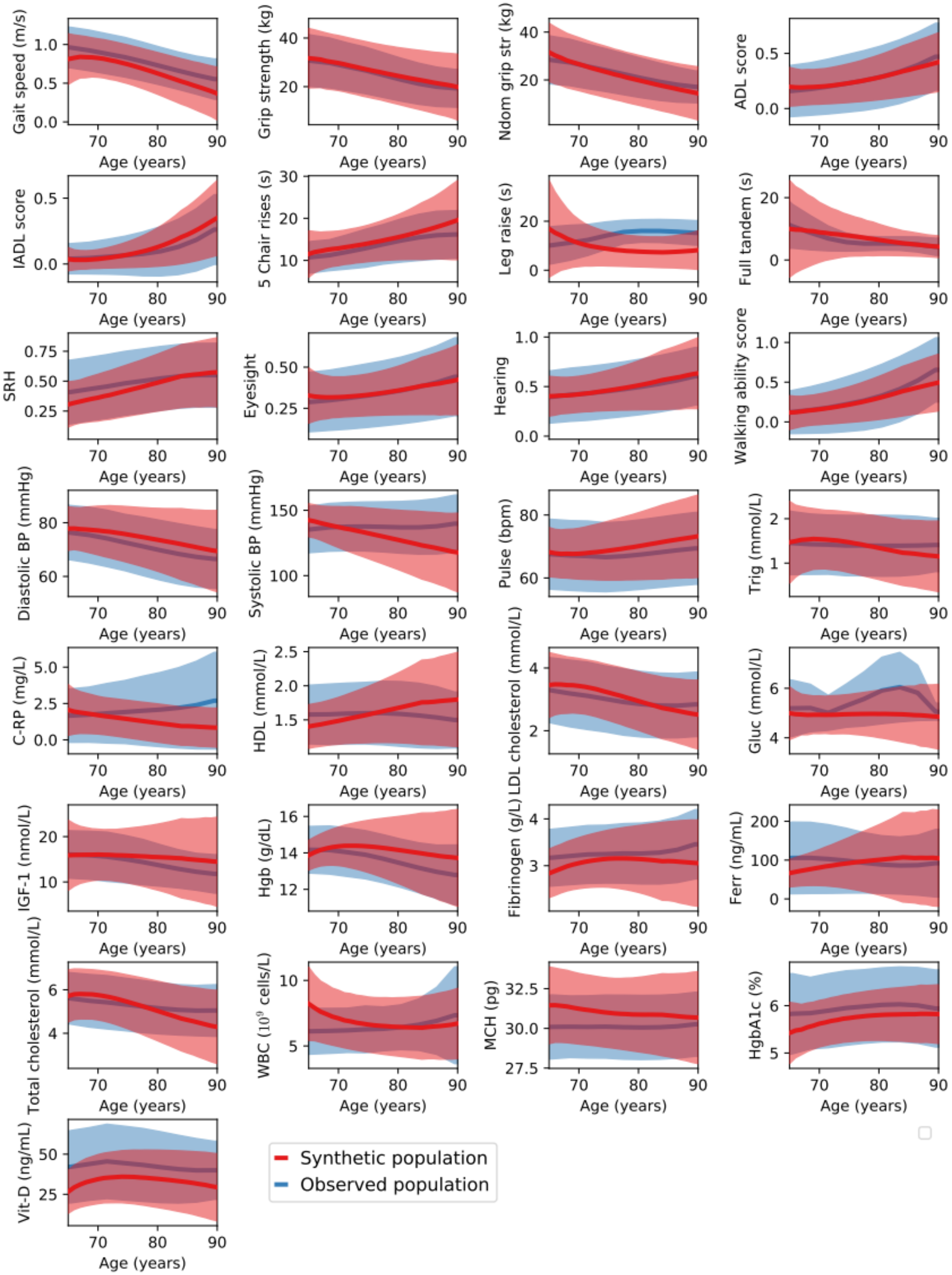

This figure shows the synthetic population generated with the DJIN model trained without individual replication. We see that standard deviations are modestly improved, although the means do not match the observed data as well as with individual replication.

2. I appreciate the detailed description of the approach and nice illustrations that provide a lot of useful information about the approach and its performance. I have a few suggestions on the presentation, which I think can further improve the readability: A) As the entire description is dispersed between different places (sections II, IV and Supplement), I would find it useful to repeat some basic details in the beginning of section IV (such as what are indices i, j, k, what are K, L, M, N, etc.) where all the equations are introduced. One example where I was lost is eq. (22)-(23) where it was not clear what is i, t, c^(m1). B) Similarly, I would introduce notations at the first place where they are used (for example, the elementwise multiplication symbol is introduced on p. 9 but first used in eq. (3); notation for the approximate posteriors (q) is explained in Suppl. but not in the main text, etc.). C) Some notations are not consistent throughout the text. For example, theta_P is in eq. (3) in mu_x, but not in the text in the 3rd line on p. 8; h_t is not in eq. (6) but it is later introduced in eq. (21) in IV.E.

A) We have added indexes to clarify throughout the methods.

B) We have introduced the definition of element-wise multiplication earlier. We have indicated our notation with q before using it.

C) We have fixed the use of theta. h_t does not appear in equation 6, because the way it is written, lambda is simply a function of {x_t}_t, u_{t_0}, and t. However the introduction of h does appear in the text after the equations.

3. In Results "population means" are mentioned on a few occasions. As those come from the training set, as I understand, I'd argue that it's not a population mean, strictly speaking, as this is coming from a subsample of the entire study. Sample means is a better term here in my opinion.

We agree, this has been changed.

4. Some variables used in the model are discrete ordinal variables with just a few categories. Arguably, the predictions/approximations of those are worse than the continuous ones. Could you elaborate on some recommendations what would be the minimal number of distinct categories for ordinal variables (and the proportion of such variables among all variables) for which the algorithm still gives reasonable results? Say, if one has 10 binary variables, 10 ordinal and 1 continuous, would you still recommend using your method in this case? I also wonder if you check if using only continuous variables from your list improves the performance of the method.

Some of the discrete ordinal variables are well predicted, for example ADL score, walking ability, IADL score, and self-rated health are all discrete and in the best half of predicted variables. The synthetic generated distributions of discrete variables also look reasonable compared to the observed sample data in Supplemental Figure 6. Additionally, some of the worst predicted are continuous, such as vitamin D, fibrinogen, C-reactive protein, leg raise, and full tandem stance.

All variables here were assumed continuous and modelled with a Gaussian likelihood for simplicity, but it is possible to change the likelihood for some of the variables, or apply transformations to make the variables better fit a Gaussian likelihood. For binary variables, we could use a Bernoulli likelihood with a sigmoid transformation. For discrete ordinal variables we could use a modified logit transformation, log{(x-min[x])/(max[x]- x)}, to transform

bounded ordinal variables to real numbers. Alternatively, we can use an ordered logit model for discrete ordinal variables with few categories.

We do not see any fundamental issues with predicting ordinal or binary variables, but we have not systematically explored this yet since any such extension requires re-optimization of the model architecture and hyperparameters.

5. Section IV.A mentions that "Ages and death ages for individuals above age 90 are also imputed this way in addition to the year that the wave occurred." Can you elaborate more on imputation of ages at death? Imputing ages at death is usually not a trivial exercise. Death can occur at any time between the waves. 1-2 years can make a big difference for the ages above 90.

Some individuals start with ages below 90, but then have missing ages above 90 when ages are privatized. We fill in these ages above 90 by assuming differences of 2 years between the waves, e.g. an individual with measured ages 87, 89, <missing>, <missing>, will be deprivatized as 87, 89, 91, 93. If we know that an individual died, this same process is done for death ages, otherwise we do it for the last age known alive for censored individuals. We do not attempt to impute death ages for censored individuals. Censoring (don't know wave of death) and privatization (know wave of death) are distinct.

We believe some of the confusion here may be due to the term "imputation". We have replaced this with "deprivatize". This does introduce the assumption of 2 year intervals between waves, which is taken from the design of the study. We have been clearer about this in our Methods. We have added Supplemental figure 15 demonstrating the accuracy of this method on known ages below 90, where we find the average error to be only 0.23 years.

6. In the same section, it is said "Individuals with no recorded death age are considered censored at their last observed age." It is not clear as the text above suggests that ages at death were imputed. Please clarify.

As in comment 5., we do not attempt to impute censored death ages. We only deprivatize death ages that are missing due to the privatization above 90, by assuming 2 year intervals between waves.

7. As this algorithm includes both generating trajectories of health states and mortality, I assume the trajectories need to be stopped at the time when the event (mortality) happens for a particular individual. It is not clear from the description whether/how this was implemented in the algorithm.

When generating trajectories we use the mortality rate to sample the probability of dying in the next time-step. We have clarified this in the paper when discussing the model equations in the methods.

8. There is inconsistent notation in the text and Suppl. (e.g., Suppl. Table 1 vs. Table I; Suppl. Fig. 1 vs. Fig. S1; "Supplemental Table 2" in IV.F should be Suppl. Table III; in title of Suppl. Table III – "Sec. III of the methods" but there is no Sec. III in the Methods section of the paper).

We have changed these.

9. Suppl. Table III is in the middle of Suppl. Figures section. I think it could be moved up to the other suppl. tables, for consistency. In addition, I would find it more convenient if the supplementary figures are reordered to match how they are cited in the text. Otherwise, the readers need to scroll up and down.

We have fixed Table III location and reordered supplemental figures.

10. The text would benefit from an additional proofreading, as there are a few typos. Here is what I caught: "heath" in Abstract and in the Last line on p. 8, "when the when the" in Fig. 2 and Fig. S12; "with with" on p. 4; "that that" on p. 5; "allows us to use add self-supervision" in IV.B.

We have have fixed these issues as well as others we found on additional proofreading.

<u>Reviewer 2:</u>

First, the authors have chosen 29 health variables from the ELSA study claiming that their choice was dictated "by availability, but not predictive quality". However, this choice looks very arbitrary with many if not all chosen variables strongly dependent on each other as, for example, leg raise and walking ability. Many variables are in fact a subset of other variables as, for example, walking ability, ADL and IADL scores, or self-rated health and hearing. Hence the chosen variables do not form an independent 29-multidimensional basis, but perhaps can be described in a drastically reduced and much simpler space.

We have included a new figure in the paper (Figure 3) that shows the performance of our DJIN model vs latent variable models with a reduced number of dimensions. This plot shows that high-dimensional models are required for predicting health outcomes, although lower dimensional models can be used for survival. We have added the methods for this to the methods section of the paper, and moved the description of the "full neural network drift" and "1D summary" models from the supplemental to this section as well.

While we agree that there are strong correlations between some of our variables (see Supplemental Fig 14a), these do not appear to drastically reduce the model dimensionality needed to predict trajectories. Precisely how the dimensionality of the latent space scales with the number of health variables, and how that depends on correlations between health variables, are both interesting questions that we have not systematically explored. We simply used what data was available in the ELSA study.

Second, pair-wise interactions between different variables are assumed to be linear. This is solely done for the purpose of claiming interpretability, since it is straightforward to apply interpretation to a linear weighting. However, since many variables are interdependent (see above) the interpretation is mostly trivial as, for example, connection between levels of glucose and glycated hemoglobin.

Despite the simplicity of the linear interactions, we show that it works well in comparison to non-linear latent-space models in the new Figure 3.

One reason this linear pair-wise network approximation may work well is because we are interested in long term predictions, rather than short-time scale dynamics where the variables are likely more strongly non-linearly coupled. Predictions with non-linear models may therefore be better with continuously tracked data, but the linear-interaction approximation of the DJIN model works well for the data we considered. It will be interesting to see how well this approach generalizes to other datasets, and the nonlinear latent space models represent an alternative approach if it does not.

Third, some of the chosen variables are sparsely represented in the observed population and are imputed using the stochastic approach. While linear pair-wise relationships derived by the model are interpretable, it is not clear how level of confidence can be assigned to this interpretation. For example, one of the strongest weights in Fig. 3 is that Vitamin D has a negative connection weight with self-rated health. While perhaps an interesting finding, however Vit D measurements are the sparsest of all (as seen from Fig.S1). Imputed VitD distribution within synthetic population is also very far from being close to the observed (Fig.S6).

Our approach does allow us to compute approximate posterior credible intervals, although they will underestimate the true size of those intervals (see our first response to the first referee above). We use these approximate credible intervals to construct the network heatmap in Figure 4 by dropping all weights with 99% credible intervals excluding zero.

We have also added a plot (supplemental Fig 13) with multiple fits of the DJIN model to show that inferred networks are robust between fits. In particular, the connection you have pointed out between vitamin D and self reported health appears to not be robust between fits. We believe it is best to interpret the network as explaining which connections the model is using to make predictions, rather than inferring biological mechanisms. While it may infer biological mechanisms, these would need testing with e.g. intervention studies. The model therefore can guide suggestions for which interventions would be interesting to study.

Fourth, while generating synthetic dataset using the same "imputation" approach that is used to augment the sparse baseline data is perhaps justifiable as a method for sanity checking, however, because of the interdependence of the variables, the observation that the sampled data does "look like" the actual data and, hence, it is properly representing how different variables impact aging is not well grounded. Fifth, the synthetic dataset is claimed to be useful for training further models when real data are not available. However, this dataset will carry only linear dependencies between interdependent variables that the model learned and, therefore, will be deprived of all other perhaps more interesting interactions.

We have included the high-dimensional latent variable model to Supplemental Figure 5, where we evaluate the quality of generated synthetic populations. We have shown the linear pair-wise network DJIN model is similar or better than high-dimensional latent variable models that include non-linear interactions.

Our purpose in providing synthetic data is to help develop competing or complementary approaches. We agree that the data is limited to be consistent with our DJIN model, but we hope it may nevertheless be useful.
